# Draft genome assemblies of doxycycline-resistant derivatives of *Bacillus cereus* strain ATCC14579

**DOI:** 10.1128/mra.01259-25

**Published:** 2026-04-27

**Authors:** Sarah Harrison, Amanda Ernlund, Kathleen Verratti, Timothy E. Long, Surya Teja Naidu, Ellen McRae Greytak, Bryan Necciai, Shanmuga Sozhamannan, Rachael Sparklin

**Affiliations:** 1 Johns Hopkins University Applied Physics Laboratory 70603, Laurel, Maryland, USA; 2 Department of Pharmaceutical Sciences, School of Pharmacy, Marshall University 505630, Huntington, West Virginia, USA; 3 Parabon NanoLabs, Inc. 344556 , Reston, Virginia, USA; 4 Capability Program Executive for Chemical, Biological, Radiological and Nuclear Defense (CPE-CBRND), Joint Project Lead for CBRND Enabling Biotechnologies, Fort Detrick, Maryland, USA; 5 Joint Research and Development, Inc., Stafford, Virginia, USA; Fluxus Inc., Sunnyvale, California, USA

**Keywords:** antimicrobial resistance, *Bacillus cereus*, One Health, doxycycline, whole-genome sequencing

## Abstract

Doxycycline resistance in *Bacillus cereus* involves diverse and understudied mechanisms. Here, we present draft genome assemblies of 104 experimentally evolved *Bacillus cereus* strains that exhibit increased growth in the presence of doxycycline, many containing novel mutations not previously described for this phenotype.

## ANNOUNCEMENT

*Bacillus cereus* is a spore-forming, rod-shaped, gram-positive bacterium that can exist as a commensal or pathogenic organism in humans. *B. cereus* can cause severe infections in the eyes, lungs, and central nervous system and is intrinsically resistant to β-lactam antibiotics, often leading to infections being treated with ciprofloxacin or vancomycin (1). However, the inevitable rise of antibiotic resistance highlights the need to understand its mechanisms to improve monitoring, prediction, and mitigation of future outbreaks (2).

To elucidate genotypic changes associated with doxycycline resistance, we performed the experimental evolution of doxycycline-resistant strains of *B. cereus*. The susceptible parent strain American Type Culture Collection (ATCC) 14579 was subjected to 20 rounds of doxycycline exposure in cation-adjusted Mueller Hinton (MH) broth (CAMHB). Exposure occurred in a 96-well plate with doxycycline concentrations ranging 0.0625–32 µg/mL. The plate was covered and incubated for 22–24 h at 37°C in a humidified incubator. After 24 h, wells were inspected for growth, and the modal minimum inhibitory concentration (MIC) was recorded. We then selected the culture at 0.5× MIC at each round for re-exposure. Following each selection cycle, single colonies were isolated from the cultures by plating, and MICs were determined by the microdilution assay in accordance with Clinical and Laboratory Standards Institute (CLSI) M0-A10 protocols for antibiotic testing of aerobic bacteria. Genomic sequencing was performed on the resulting mutants.

For DNA preparation, bacterial strains were recovered from glycerol stocks by plating on MH agar and incubating overnight at 37°C. Single colonies were transferred to 50 mL of MH broth and grown overnight at 37°C with shaking at 180 rpm. The culture was centrifuged, and the pellet was used for DNA extraction using Qiacube QIAamp Mini Bacterial or Yeast DNA with Enzymatic Lysis V1 Protocol. Enzymatic lysis was performed in 180 ul TE buffer, 1.2% Triton, and 20 mg/mL lysozyme. Quality and quantity of extracted DNA were assessed using the NanoDrop.

Sequencing libraries were prepared by Plasmidsaurus. DNA was prepared using the Rapid ONT Barcoding Kit (SQK-RBK114.96), then sequenced on GridION using flow-cell R10.4.1. Illumina libraries were prepared using the Illumina DNA Prep Kit (20060059), and sequencing was performed on the NovaSeq X, generating paired-end 2 × 150 bp reads. Plasmidsaurus hybrid assembly pipelines can be found here: https://plasmidsaurus.com/technical-documentation/genome; but briefly, basecalling, demultiplexing, and adapter trimming for Nanopore data were performed using Dorado v0.5.2 (3) with the super high accuracy model. Additional QC filtering was performed using FiltLong 0.2.1 (4). Illumina quality filters were used for QC, and adapter sequences were removed from the Illumina data using fastp v0.23.2 (5). Nanopore data were assembled using the Hybracter v0.11.0 pipeline, including using Flye v2.9.6+ (6) and Hifiasm with one round of long-read polishing using Medaka v1.8.0 (7) polished using Polypolish v0.6.0 (8). Plasmids were assembled using Plassembler v1.8.0 (9). The hybrid genome was annotated by National Center for Biotechnology Information (NCBI) using PGAP 6.10, and reference mapping was performed using Minimap2 (10) for the Nanopore data and BWA (11) for the Illumina data. Default parameters were used for all software, except Minimap2 (-ax for long-read data). Assessment using CheckM v1.1.6 and CheckM v2 v1.0.2 estimated that all genomes were >99% complete and<3% contaminated (12).

**TABLE 1:** Genome sequence details of *B. cereus* doxycycline-exposed derivatives^*a*^

			Illumina data		ONT data										
Abx exp. no.	Modal MIC (ug/mL)	Sample no.	SRA accession no.	Total reads	SRA accession no.	Total reads	ONT N50	GenBank accession	Assembly size (bp)	No. of contigs	GC content	Assembly N50	No. of CDSs	Depth of coverage (X)	Breadth of coverage
D4	4	D4-1, D4-2, D4-3, D4-4, D4-5, D4-6	SRR33148381, SRR33148383-SRR33148387	1,179, 160.3 ± 609, 819.6	SRR33123820, SRR33123822-SRR33123826	176, 113.7 ± 150, 862.7	6,044.7 ± 20,376.0	GCA_055690355.1, GCA_055690345.1, GCA_051390095.1, GCA_051390055.1, GCA_051390035.1, GCA_055690335.1	5,262, 395.8 ± 197, 491.4	4.2 ± 2.0	35.4 ± 0.1	4,472, 545 ± 1, 046, 898	5,292 ± 140	75.0 ± 35.1	96.5 ± 3.6
D5	4	D5-1, D5-2, D5-3, D5-4, D5-5, D5-7, D5-8, D5-9, D5-10, D5-11, D5-12, D5-13, D5-14, D5-15, D5-16, D5-17, D5-18, D5-19, D5-20, D5-21, D5-22, D5-23, D5-24, D5-25, D5-26	SRR33148319-SRR33148324, SRR33148336-SRR33148342, SRR33148344-SRR33148346, SRR33148371-SRR33148373, SRR33148375-SRR33148380	1,225, 462.5 ± 682, 669.8	SRR33123793-SRR33123798, SRR33123800-SRR33123809, SRR33123811-SRR33123819	331, 524.8 ± 427, 192.1	37,364.6 ± 20,843.4	GCA_055690325.1, GCA_055689815.1, GCA_055689675.1, GCA_051389955.1, GCA_051389975.1, GCA_055798455.1, GCA_055798725.1, GCA_051201745.1, GCA_052659125.1, GCA_055690225.1, GCA_055690215.1, GCA_051390015.1, GCA_055689975.1, GCA_055798445.1, GCA_055689855.1, GCA_055798335.1, GCA_055689835.1, GCA_055689825.1, GCA_055689805.1, GCA_051389995.1, GCA_052658985.1, GCA_055798325.1, GCA_055798075.1, GCA_055689695.1, GCA_055798085.1	5,404, 337.0 ± 98, 787.2	3.3 ± 2.3	35.3 ± 0.1	5,033, 264 ± 998, 335	5,394.7 ± 140.3	75.8 ± 34.6	99.2 ± 1.8
D6	16	D6-1, D6-2, D6-3, D6-4, D6-5, D6-6, D6-7, D6-9, D6-10, D6-11, D6-12, D6-13, D6-14, D6-15	SRR33148325-SRR33148328, SRR33148330-SRR33148335, SRR33148350, SRR33148370	1,232, 429.9 ± 502, 527.4	SRR33123780-SRR33123787, SRR33123789-SRR33123792	310, 688.5 ± 325, 245.8	45,556 .4 ± 10,179 .1	GCA_051389935.1, GCA_051389855.1, GCA_051389815.1, GCA_051389795.1, GCA_051389775.1, GCA_051389755.1, GCA_051389715.1, GCA_051389735.1, GCA_051389895.1, GCA_051389915.1, GCA_051389075.1, GCA_051389055.1, GCA_051389875.1, GCA_051389835.1	4,710, 666.6 ± 1,481, 366.9	4.8 ± 1.7	35.7 ± 1.0	3,213, 553 ± 1,335, 856	5,102 ± 209	70.8 ± 38.6	94.3 ± 1.3
D7	16	D7-1, D7-2, D7-3, D7-4, D7-5, D7-6, D7-7, D7-8, D7-9, D7-10, D7-11, D7-12, D7-13, D7-14, D7-15, D7-16, D7-17, D7-18, D7-19, D7-20, D7-21, D7-22, D7-23, D7-25, D7-26, D7-27, D7-28, D7-29, D7-30, D7-31	SRR33148308-SRR33148310, SRR33148315-SRR33148318, SRR33148348-SRR33148349, SRR33148351-SRR33148357, SRR33148359-SRR33148368	1,157, 363.1 ± 693, 876.5	SRR33123751-SRR33123765, SRR33123767-SRR33123776, SRR33123778-SRR33123779	558, 639.5 ± 587, 038.5	29,147 .7 ± 6,421 .0	GCA_051389695.1, GCA_051389495.1, GCA_051389315.1, GCA_051389255.1, GCA_051389215.1, GCA_051389235.1, GCA_051389095.1, GCA_051389155.1, GCA_051388995.1, GCA_051389675.1, GCA_051389655.1, GCA_051389635.1, GCA_051389615.1, GCA_051389595.1, GCA_051389575.1, GCA_051389555.1, GCA_051389535.1, GCA_051389515.1, GCA_051389015.1, GCA_051389035.1, GCA_051389475.1, GCA_051389455.1, GCA_051389435.1, GCA_051389415.1, GCA_051389395.1, GCA_051389375.1, GCA_051389355.1, GCA_051389335.1, GCA_051389275.1, GCA_051389295.1	5,089, 889.0 ± 89, 143.9	6.3 ± 3.2	35.4 ± 0.1	3,291, 471 ± 888, 883	5,039 ± 361	64.9 ± 35.1	93.7 ± 1.6
D9	32	D9-1, D9-2, D9-3, D9-4	SRR33148311-SRR33148314	226, 965.8 ± 89, 052.6	SRR33123747-SRR33123750	114, 014.3 ± 78,966 .6	11,609 .5 ± 5,310 .7	GCA_051389195.1, GCA_051389175.1, GCA_051389135.1, GCA_051389115.1	5,064, 585.0 ± 8, 712.5	6.5 ± 3.0	35.4 ± 0.1	2,696, 107 ± 1,069, 995	5,081.3 ± 4.6	9.0 ± 3.5	92.9 ± 0.2
D12	32	D12-1, D12-2, D12-3, D12-4, D12-5, D12-6	SRR33148343, SRR33148374, SRR33148382, SRR33148393, SRR33148404, SRR33148405	349,970 .0 ± 163, 109.8	SRR33123799, SRR33123810, SRR33123821, SRR33123832, SRR33123843, SRR33123844	1,301, 858.3 ± 469, 469.1	14,928 .0 ± 2,625 .6	GCA_051390575.1, GCA_051390555.1, GCA_051390515.1, GCA_051390535.1, GCA_051390495.1, GCA_051390455.1	5,048, 056.5 ± 22,869 .6	4.8 ± 0.8	35.4 ± 0.0	3,231, 104 ± 2.0	5,058.8 ± 22.2	29.4 ± 9.3	92.6 ± 0.4
D15	16	D15-1, D15-2, D15-3, D15-4, D15-5, D15-6, D15-7, D15-8	SRR33148400-SRR33148403, SRR33148329, SRR33148347, SRR33148358, SRR33148369	394, 044.0 ± 212, 101.3	SRR33123755, SRR33123766, SRR33123777, SRR33123788, SRR33123839- SRR33123842	995, 847.0 ± 358, 298.1	12, 961.5 ± 2,584 .8	GCA_051390475.1, GCA_051390435.1, GCA_051390415.1, GCA_051390395.1, GCA_051390375.1, GCA_051390355.1, GCA_051390315.1, GCA_051390335.1	4,938, 849.1 ± 177, 554.0	4.8 ± 0.9	35.5 ± 0.1	3,230, 612 ± 913	4,947 .1 ± 172 .7	28.3 ± 9.5	90.6 ± 3.3
D18	32	D18-1, D18-2, D18-3, D18-4, D18-5	SRR33148395- SRR33148399	333, 902.8 ± 140, 556.7	SRR33123834- SRR33123838	78,245 .2 ± 40,137 .2	14,363 .0 ± 4,765 .2	GCA_051390295.1, GCA_051390255.1, GCA_051390275.1, GCA_051390235.1, GCA_051390215.1	468,496. 6 ± 46, 867.5	6.8 ± 3.7	35.5 ± 0.0	3,240, 914 ± 12,090	4,704.0 ± 50.4	16.6 ± 10.3	85.9 ± 0.9
D19	32	D19-1, D19-2, D19-3, D19-4, D19-5, D19-6	SRR33148388- SRR33148392, SRR33148394	1,001, 652.3 ± 403, 403.8	SRR33123827- SRR33123831, SRR33123833	227, 843.7 ± 39,521 .6	41,821 .7 ± 4,034 .7	GCA_051390195.1, GCA_051390175.1, GCA_051390155.1, GCA_051390135.1, GCA_051390115.1, GCA_051390075.1	4,842, 413.8 ± 171, 415.1	5.5 ± 1.4	35.5 ± 0.0	3,245, 699 ± 20, 710	4,809 ± 147	86 ± 36.6	88.8 ± 3.1

^*a*^ Antibiotic exposure number (C# = exposure passage number), modal MIC for all colonies sequenced, the number of colonies from each exposure (sample no), and sequencing statistics are provided. Strains are grouped by passage number for clarity and conciseness.

## Data Availability

The accession numbers and sequence statistics of the 104 genomes across the doxycycline re-exposure cultures are listed in Table 1. Data are deposited in NCBI SRA under BioProject accession number PRJNA1244367 and BioSample accessions and assembled genomes in GenBank under accessions denoted in Table 1.
